# Optical and electrical features of semitransparent CuO photoelectrochemical cell

**DOI:** 10.1016/j.dib.2018.01.074

**Published:** 2018-02-01

**Authors:** Malkeshkumar Patel, Joondong Kim

**Affiliations:** a Photoelectric and Energy Device Application Lab (PEDAL), Multidisciplinary Core Institute for Future Energies (MCIFE), Incheon National University, 119 Academy Rd. Yeonsu, Incheon 22012, Republic of Korea; b Department of Electrical Engineering, Incheon National University, 119 Academy Rd. Yeonsu, Incheon 22012, Republic of Korea

## Abstract

The data presented in this article are related to the research article entitled “CuO photocathode-embedded semitransparent photoelectrochemical cell” (Patel et al., 2016) [1]. This article describes the growth of Cu oxides films using reactive sputtering and application of CuO photocathode in semitransparent photoelectrochemical cell (PEC). In this data article, physical, optical and electrical properties, and PEC performances data set of the reactively sputtered semitransparent CuO samples are made publicly available to enable extended analyses.

## Specifications table

**Table:** 

Subject area	*Materials Engineering, Physics, Electrochemistry*
More specific subject area	*Solar Energy*
Type of data	*Figures, Table*
How data was acquired	*Field emission scanning electron microscope (FESEM; JSM-7800F, JEOL Ltd., Tokyo, Japan) Surface profiler (Dektak XT-E, Veeco, Plainview, New York)*
	*UV-visible spectrophotometer (UV-2600, Shimadzu Corporation, Seoul, South Korea),*
	*Potentiostat/Galvanostat (ZIVE SP1, WonA Tech, Korea)*
	*PEC cells (Copper oxide-coated FTO, Ag/AgCl, and platinum gauze were connected to the working, reference, and counter electrodes of the PG-stat, respectively, Aqueous 0.1 M NaOH solution was used as electrolyte)*
Data format	*Analyzed*
Experimental factors	*Prepared CuO samples were treated under rapid thermal processing to observe the morphologies before and after air annealing*
	*Surface profiler: contact mode, scanning length 2 mm, and force 5 mg.*
	*Optical Reflectance: CuO photocathode on glass substrate*
	*Mott–Schottky: Frequency*→*500 Hz to 5 kHz*
	*Bias range*→*0.4 V to − 0.6 V vs. Ag/AgCl*
Experimental features	*Phase and stoichiometry tunable growth of CuO samples using the reactive sputtering of Cu target, and application in semitransparent photocathode*
Data source location	*Incheon National University, Incheon-406772, Korea*
Data accessibility	*The data are with this article*

## Value of the data

• Performance comparisons of semitransparent CuO photocathode to other Cu oxides (CuO_x_) based materials. This comparison includes photoelectrochemical (PEC) cell measurement and performance parameters, such as band gaps of CuO materials, types of the electrolyte, light sources, photocurrent density, and methods of CuO fabrication. Readers can easily summarize the progress of CuO PEC cells.
• Approaches to modulate the morphologies of CuO_x_ films. A simple and powerful reactive sputtering method can be applied to tune the CuO_x_ films. The surface morphology of various Cu oxides was obtained by changing the oxygen flow rate during the sputtering process.
• RTP effect is significant and effective to enhance the crystallinity of CuO_x_ films.
• The Mott–Schottky analysis confirms the modulation of built-in potential of CuO_x_ films.
• Tuning of the optical band gap of semitransparent CuO photocathode would be useful for bandgap engineering and applied for advanced CuO embedded photoelectrochemical cells.

## 1 Data

**Fig. 1:**
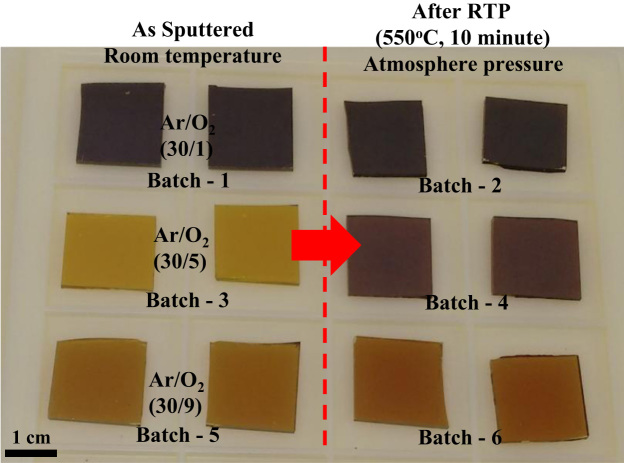
Photograph of samples including their classification and process conditions.

**Fig. 2:**
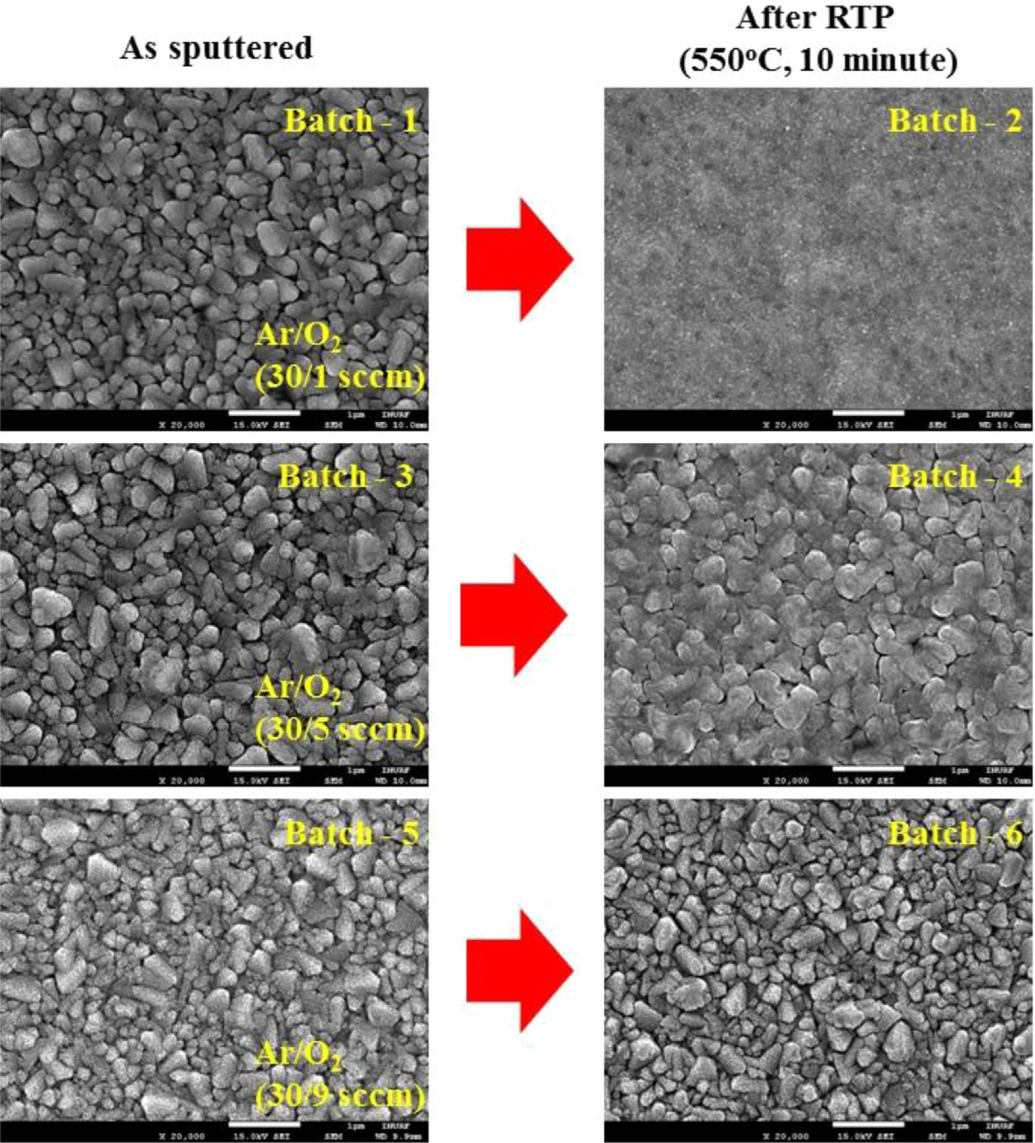
SEM images of the samples featuring the surface morphological variation for given synthesis condition. Left images presents as sputtered samples, the surface morphology of various Cu oxides prepared by changing the oxygen flow rate. Right images presents samples treated by atmospheric RTP, the surface morphology of nanoscaled CuO converted from various Cu oxides. Scale bar, 1 μm.

**Fig. 3:**
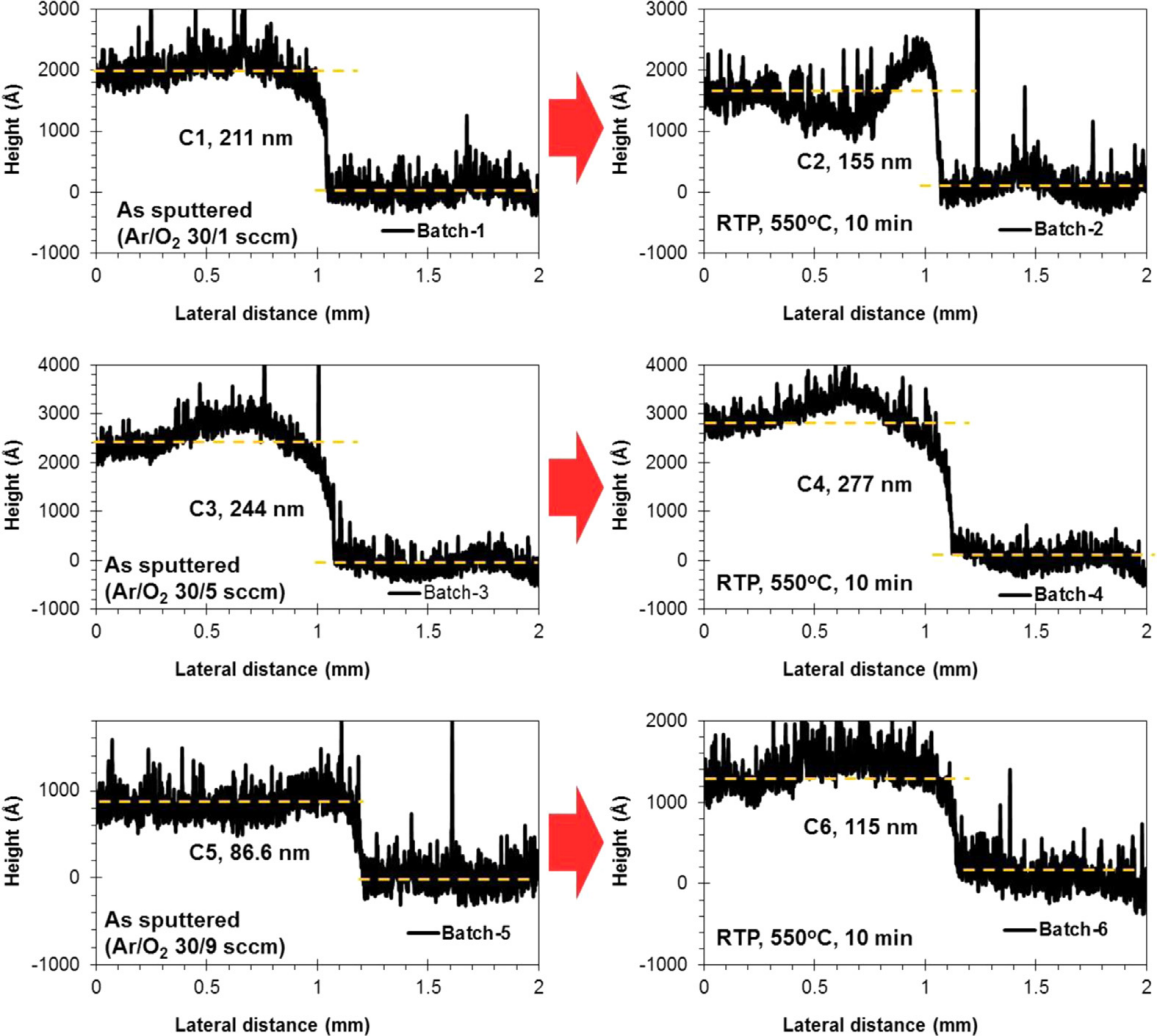
Depth profiles of batch samples. The estimated thin film thickness, process parameters of the samples are marked in each plot.

**Fig. 4:**
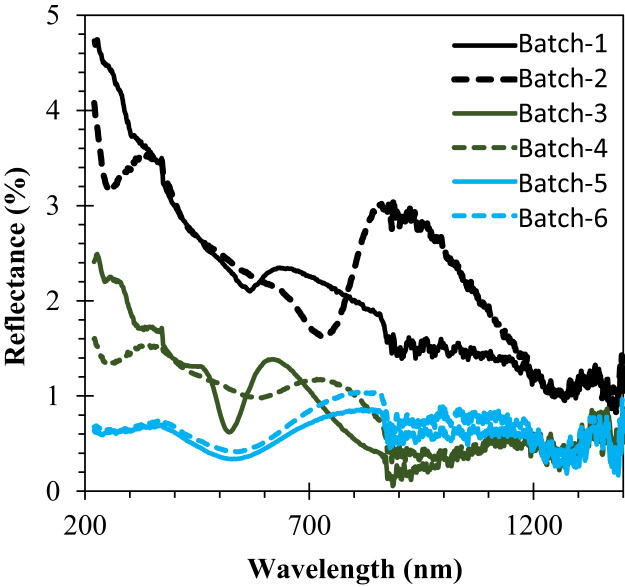
Reflectance profiles of as sputtered and RTP treated samples.

**Fig. 5:**
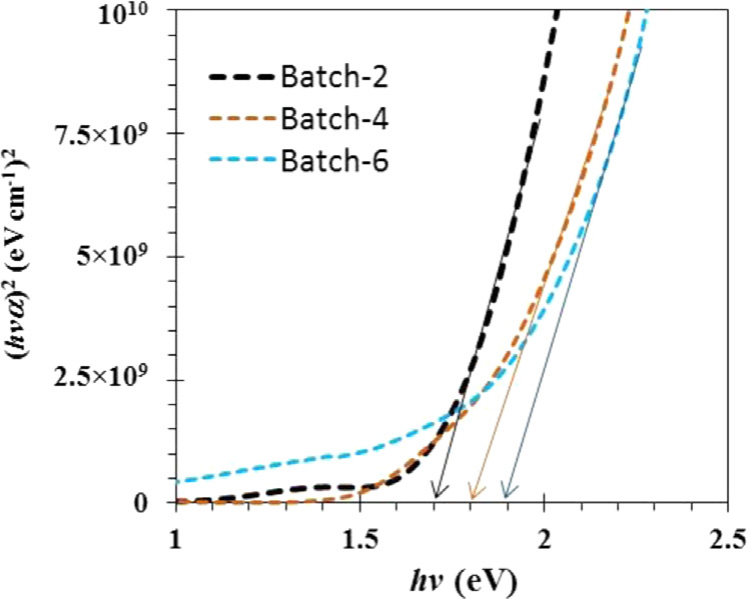
Tauc plot of RTP-treated samples.

**Fig. 6:**
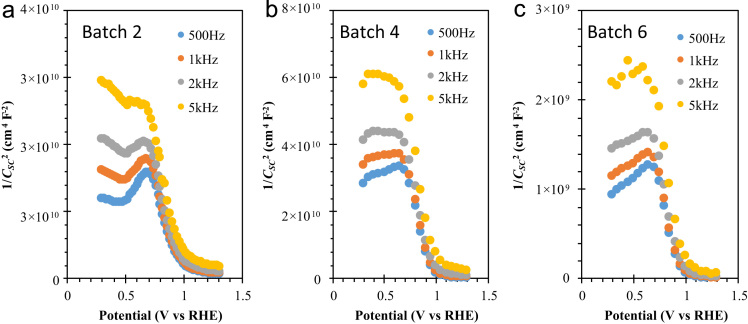
Mott–Schottky plots of samples treated by RTP (a) Batch 2, (b) Batch 4 and (c) Batch 6. These samples present various nanoscale features of CuO materials. Here, 1/*C*^2^ vs. *V* shown for various frequencies from 500 Hz to 5 kHz. Consistence slope and intersection on potential axis firmed the accurate accepter carrier concentration and flat band potentials of these samples are attributed to the bulk properties and without involving surface states.

**Fig. 7:**
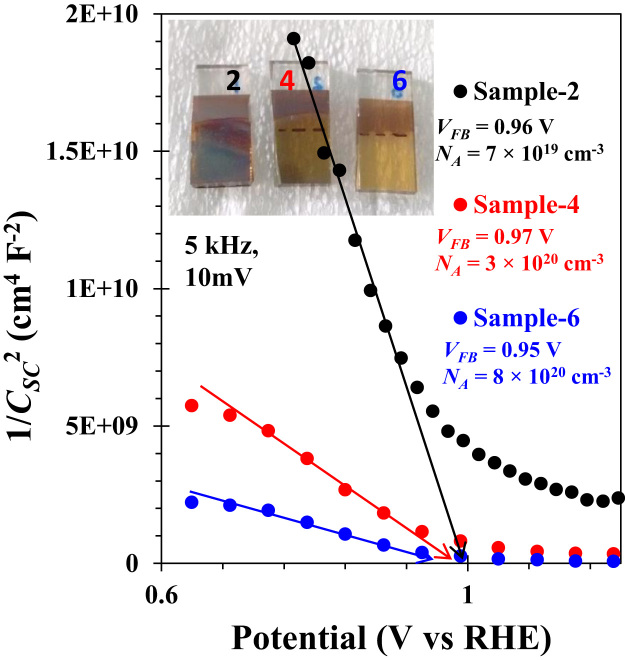
Estimated accepter carrier concentration (*N*_*A*_) and flat band potential (*V*_*FB*_) from Mott–Schottky analysis of the samples treated by RTP.

The comparison for the reactive-sputtered semitransparent CuO photocathode was presented in Table 1. This summary is presented in the chronological order. The readers can easily overview the progress of Cu oxide-based PEC cells. Fig. 1 shows the photographs of the reactive sputtered CuO_x_ films and images after RTP. The surface morphologies of the various Cu oxides grew by various oxygen flows and RTP treatments are presented in Fig. 2. The film thickness measured using the surface profiler is presented in Fig. 3. Reflectance characteristics and Tauc plots of CuO samples are presented in Figs. 4 and 5, respectively. Frequency dependent Mott–Schottky measurement of RTP-treated CuO samples is presented in Fig. 6. Fig. 7 shows the free carrier concentrations and flat band potential of CuO samples according to the Mott–Schottky analysis.

**Table 1:** Performance comparison for our nanoscaled CuO photocathode with the CuO based photocathodes in literature. Reference potential for measured photocurrent density is mentioned as reversible hydrogen electrode (RHE), silver/silver chloride (Ag/AgCl) and saturated calomel electrode (SCE). Photocurrent value given in this work is the average value of total 3 electrodes.

**Method of preparing CuO**	***E***_***g***_**(eV)**	**Electrolyte**	**Light source**	**Photocurrent density (mA cm**^**−2**^**)**	**Year/Ref.**
Reactive DC sputtering, room temperature	1.85	0.1 M NaOH	LED, 100 mW cm^-2^	1.75 (0.3 V vs. RHE)	This work
Reactive DC sputtered+RTP	1.7	0.1 M NaOH	LED, 100 mW cm^−2^	6.4 (0.3 V vs. RHE)	This work
Grind powder+LiNO_3_	1.35	0.1 M Na_2_HP0_4_	Xenon lamp, 810 mW cm^−2^	~ 0.44 (− 0.4 V vs. SCE)	1982/[2]
Electrodeposition	1.56	–	500 W xenon lamp	~ 0.08 (− 0.2 V vs. Ag/AgCl)	2004/[3]
Sol–gel	1.77	NaOH (pH 13)	150 W xenon arc lamp	~ 2.02 (− 0.5 V vs. SCE)	2009/[4]
Electrochemical two stage growth	–	NaOH (pH 11)	W-halogen lamp, 125 mW cm^−2^	~ 0.35 (0.05 V vs. RHE)	2010/[5]
Spin coating of CuO particle prepared by flame spray pyrolysis	1.44	1 M KOH (pH 14)	1 sun	1.2 (− 0.55 V vs. Ag/AgCl)	2011/[6]
RF sputtering of CuO	–	1 M KOH (pH 14)	150 W solar simulator	~ 3.15 (− 0.55 V vs. Ag/AgCl)	2012/[7]
Flame spray pyrolysis Li:CuO	–	1 M KOH	1 sun	~ 1.69 (− 0.55 V vs. Ag/AgCl)	2012/[8]
spinning disk reaction/spin coating	1.68	1 M KOH	1 sun	1.58 (− 0.55 V vs. Ag/AgCl)	2012/[9]
Solution processed porous CuO	1.35	1 M KOH	1 sun	1.2 (− 0.55 V vs. Ag/AgCl)	2012/[10]
RF co-sputtered Cu and Ti for Ti:CuO	1.12–1.46	1 M Na_2_SO_4_	250-W quartz tungsten lamp	0.09 (− 0.5 V vs. Ag/AgCl)	2012/[11]
Sol–gel	1.2	0.1 M Na_2_SO_4_ (pH 5.84)	150 W Xenon arc lamp and AM1.5 filter	~ 0.35 (0.05 V vs. RHE)	2014/[12]
Doped CuO by flame spray pyrolysis	–	1 M KOH (pH 14)	1 sun	~ 1.07 (− 0.55 V vs. Ag/AgCl))	2014/[13]
Anodising Cu foil: TiO_2_/CuO	–	0.5 M K_2_SO_4_	300 W xenon arc lamp	2.4 (− 0.36 V vs. Ag/AgCl)	2015/[14]
Template assisted electrodeposition of CuO/ZnO	1.5	0.1 M KOH	White light	1.2 (− 0.5 V vs. Ag/AgCl)	2016/[15]
RF sputtering of CuO target	1.25	0.1 M Na_2_SO_4_ (pH 5.84)	1 sun	2.5 (0 V vs. RHE)	2016/[16]
RF sputtered CuO+RTP	1.35	0.1 M Na_2_SO_4_ (pH 5.84)	1 sun	1.68 (0 V vs. RHE)	2016/[17]
Doped Ni:CuO by flame spray pyrolysis	–	1 M KOH (pH 14)	1 sun	1.07 (− 0.55 V vs. Ag/AgCl)	2016/[18]
Spray pyrolysis+Calcination	1.57	1 M KOH (pH 13.5)	1 sun	24 (0.25 V vs. RHE)	2016/[19]
Chemical bath deposition+Calcination	1.55	0.5 M Na_2_SO_4_ (pH 6.6)	1 sun	1.3 (0 V vs. RHE)	2017/[20]

## 2 Experimental design, materials and methods

### 2.1 Preparation of CuO_x_ films

Large scale (Ø4 in.) Cu target (purity 99.99%) was reactively sputtered to form various phases of copper oxides (CuO_x_) at room temperature. The reactive gas (O_2_) and the sputtering gas (Ar) were simultaneously supplied to tune the phases CuO_x_ by changing the O_2_ flow rate (1–9 sccm) at a fixed Ar supply (30 sccm). Three types of CuO_x_ phases were achieved for Batch-1 (Ar/O_2_ of 30/1 sccm), Batch-3 (Ar/O_2_ of 30/5 sccm), and Batch-5 (Ar/O_2_ of 30/9 sccm). To control the of CuO_x_ film properties, rapid thermal process was performed for 10 min at 550 °C. The RTP-treated samples were denoted as Batch-2 (RTP-treated Batch-1), Batch-4 (RTP-treated Batch-3), and Batch-6 (RTP-treated Batch-5), respectively [1]. In order to remain a pure FTO, the Krypton tape was partially covered the FTO glass and removed after the reactive sputtering process.

### 2.2 PEC Mott–Schottky measurements

The potentiostat/galvanostat (PG-stat; ZIVE SP1, WonA Tech, Seoul, South Korea) was applied for the PEC Mott–Schottky measurements in a three electrodes cell. Copper oxide-coated FTO, Ag/AgCl, and platinum gauze were connected to the working, reference, and counter electrodes of the PG-stat, respectively. Aqueous 0.1M NaOH solution was used as an electrolyte for all PEC measurements.

The measured potential *V* vs. Ag/AgCl was converted to the reversible hydrogen electrode (RHE) scale according to the Nernst equation: *E*_RHE_ = *E*_Ag/AgCl_ + 0.059 pH + *E*_o/Ag/AgCl_, where *E*_RHE_ is the converted potential vs. RHE, *E*_o/Ag/AgCl_ = 0.1976 V at 25 °C, and E_Ag/AgCl_ is the experimentally measured potential against Ag/AgCl reference. The Mott–Schottky (1/*C*_SC_^2^ vs. *V*) analysis of photoelectrodes was performed in varying the frequencies (500 Hz to 5 kHz) with the AC signal of 10 mV.
