# Supplementary material for: Optical and electrical features of semitransparent CuO photoelectrochemical cell
Source: Data Brief. 2018 Feb 1;17:681–8. doi: 10.1016/j.dib.2018.01.074 (PMC5852286; doi:10.1016/j.dib.2018.01.074)
Supplement: Supplementary file 1 — Supplementary material. [file mmc1.docx]

January 13, 2018

Dear Editor

RE: DIB-D-17-00471R1

*Title: Optical and electrical features of semitransparent CuO photoelectrochemical cell*

Authors: Malkeshkumar Patel, and Joondong Kim

We declare that this manuscript is original, has not been reported before, and is not currently being considered elsewhere. We also confirm that there is no known conflict of interest regarding this manuscript and its publication. The manuscript has been approved by all named authors.

Sincerely yours,

Joondong Kim

--------------------------------------------------------------------

Ph. D./Professor

Department of Electrical Engineering

Incheon National University

119 Academy Rd. Yeonsu Incheon, 406772, Korea

Email: [joonkim@inchoen.ac.kr](mailto:joonkim@inchoen.ac.kr) Tel: +82-32-835-8770
